# Supplementary figures and images for: Pathogenicity Detection and Genome Analysis of Two Different Geographic Strains of BmNPV
Source: Insects. 2021 Sep 30;12(10):890. doi: 10.3390/insects12100890 (PMC8537803; doi:10.3390/insects12100890)

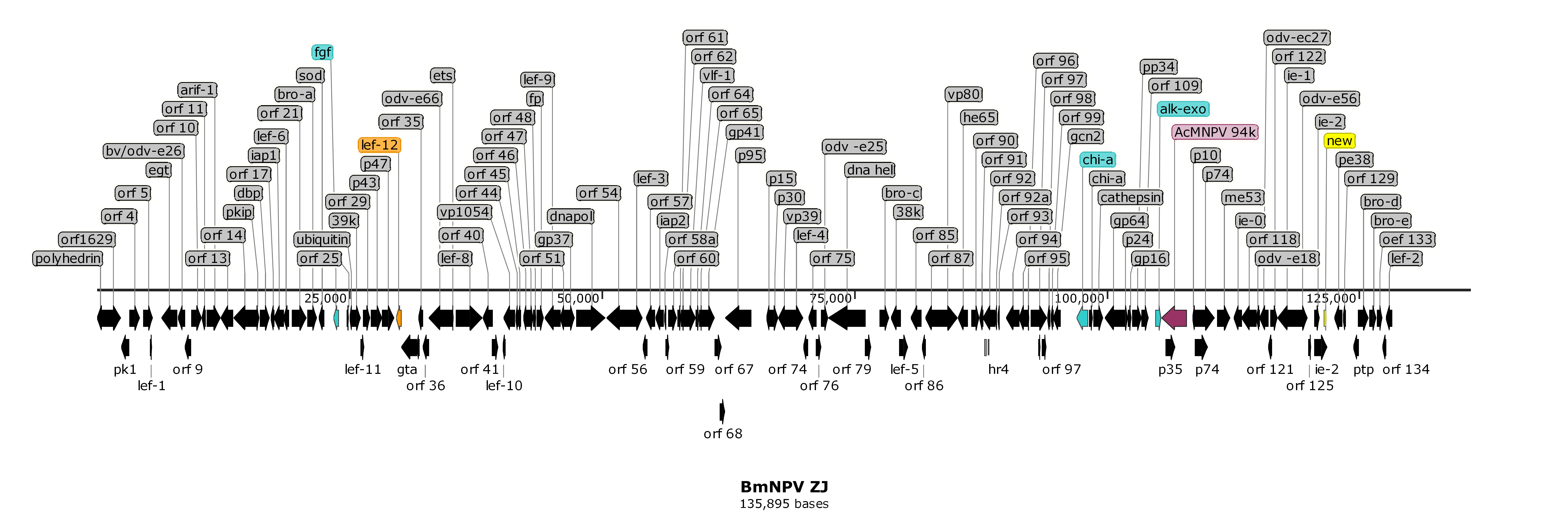

Supplement: Supplementary file 1 [file insects-12-00890-s001.zip › Figure S1 BmNPV ZJ genome linear map.tiff]

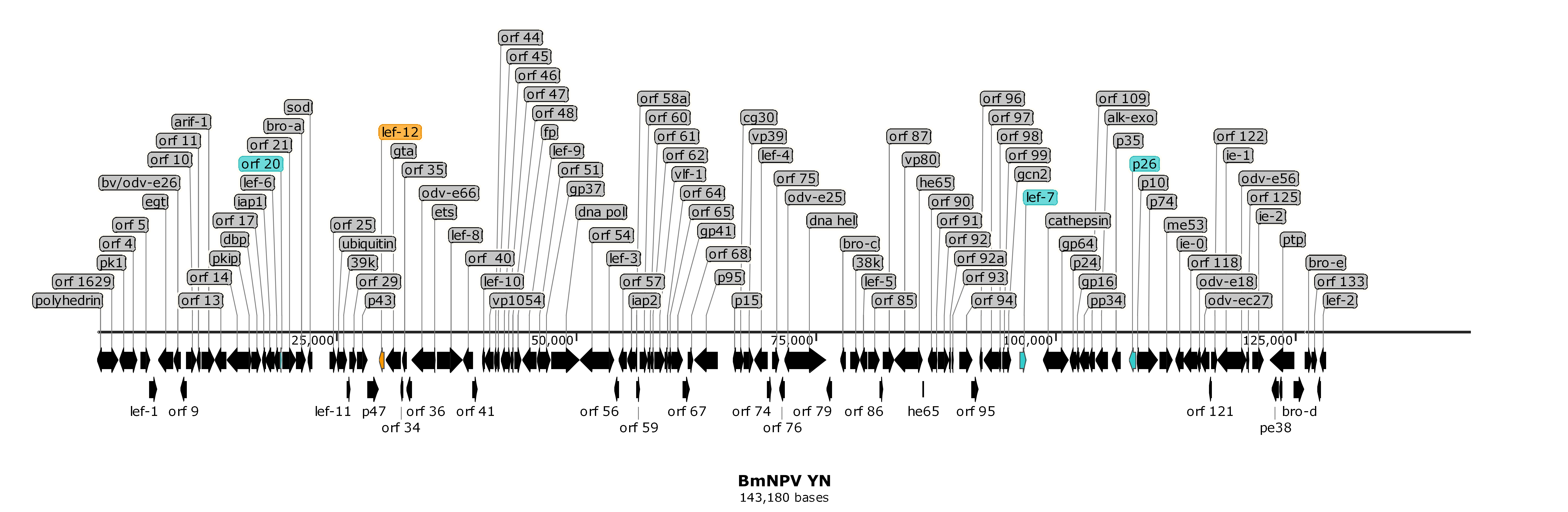

Supplement: Supplementary file 1 [file insects-12-00890-s001.zip › Figure S2 BmNPV YN genome linear map.tiff]
